# The Prognostic and Predictive Significance of Tumor-Infiltrating Memory T Cells Is Reversed in High-Risk HNSCC

**DOI:** 10.3390/cells11121960

**Published:** 2022-06-17

**Authors:** Rebekka Hartan, Sören Schnellhardt, Maike Büttner-Herold, Christoph Daniel, Arndt Hartmann, Rainer Fietkau, Luitpold Distel

**Affiliations:** 1 Department of Radiation Oncology, Universitätsklinikum Erlangen, Friedrich-Alexander-Universität Erlangen-Nürnberg, 91054 Erlangen, Germany; rebekka.h@gmx.de (R.H.); soeren.schnellhardt@fau.de (S.S.); rainer.fietkau@uk-erlangen.de (R.F.); 2 Comprehensive Cancer Center Erlangen-EMN (CCC ER-EMN), 91054 Erlangen, Germany; maike.buettner-herold@uk-erlangen.de (M.B.-H.); christoph.daniel@uk-erlangen.de (C.D.); arndt.hartmann@uk-erlangen.de (A.H.); 3 Department of Nephropathology, Institute of Pathology, Universitätsklinikum Erlangen, Friedrich-Alexander-Universität Erlangen-Nürnberg, 91054 Erlangen, Germany; 4 Institute of Pathology, Universitätsklinikum Erlangen, Friedrich-Alexander-Universität Erlangen-Nürnberg, 91054 Erlangen, Germany

**Keywords:** CD45RO, head and neck cancer, memory T cells, prognostic value, reversal of prognostic value

## Abstract

Tumor-infiltrating CD45RO+ memory T cells have unanimously been described as a positive prognostic factor in head and neck squamous cell carcinomas (HNSCCs). Here, we investigated the long-term prognostic relevance of CD45RO+ memory T cells in HNSCC with special regard to the influence of clinical characteristics. Pre-treatment biopsy samples from 306 patients with predominantly advanced HNSCC were analyzed. Immunohistochemistry was used to stain tissue microarrays for CD45RO+ memory T cells. CD45RO cell densities were semi-automatically registered and used for survival analysis. High CD45RO+ cell densities were clearly associated with prolonged overall survival (OS) and recurrence-free survival as well as no evidence of disease status after 10 years (*p* < 0.05). In contrast, the prognostic significance of tumor-infiltrating memory T cells was completely reversed in high-risk groups: in poorly differentiated tumors (G3, G4) and in cases with lymph node involvement (N+), high memory T cell densities correlated with reduced 10-year OS (*p* < 0.05). In conclusion, an increased density of tumor-infiltrating CD45RO+ cells in HNSCC can be a positive as well as a negative prognostic factor, depending on disease stage and histological grade. Therefore, if CD45RO+ cell density is to be used as a prognostic biomarker, further clinical characteristics must be considered.

## 1. Introduction

Head and neck squamous cell carcinomas (HNSCCs) are the sixth most common form of cancer worldwide, with more than 890,000 cases per year [1,2]. Despite therapeutic advances in recent years, prognosis remains poor with a 5-year relative survival rate of 65% for all stages and only 39% for patients suffering from distant metastases [3,4,5]. Therefore, the development and clinical evaluation of new therapy concepts such as the combination of immune checkpoint inhibitors with radiotherapy remains a critical challenge [6]. A profound understanding of tumor biology and tumor immunology is essential in this endeavor. It not only represents the foundation for the development of new therapeutic strategies, but also facilitates the discovery of potential prognostic and predictive biomarkers which might aid in the selection of a therapeutic regimen with maximum efficacy and minimum toxicity [2,7]. This is especially crucial in HNSCC due to the high impact of therapy-related toxicities on quality of life [8].

Current knowledge suggests that the tumor microenvironment (TME) plays a key role in tumor behavior and therapeutic response. Cellular components of the TME are, among others, cancer-associated fibroblasts, adipocytes, pericytes, mesenchymal stem cells, endothelial cells, and inflammatory cells [9]. The prognostic and predictive properties of tumor-infiltrating inflammatory cells (TIICs) have become a central focus of interest [9,10,11]. Among the many different types of inflammatory cells in the TME, tumor-infiltrating lymphocytes (TILs) in particular are most frequently studied, as their presence and location appear to be closely associated with disease outcome [12]. This work focused on the prognostic value of the TIL subset of memory T cells.

Memory T cells are commonly detected with the immune marker CD45RO [12,13,14]. CD45, also known as leukocyte common antigen, is a tyrosine phosphatase and part of the leukocyte signaling. The formation of different subforms is cell-type-specific and depends on the degree of differentiation as well as the degree of activation [13]. The CD45RO isoform is the activated subset of CD45, expressed among others on memory T cells after antigen contact. CD45RA+ cells on the other hand are not activated and are called the naive subset [12,13,15]. CD45RA+ tumor-infiltrating inflammatory cells in general are rarely investigated for their prognostic significance in malignancies. The subgroup of CD45RA+FoxP3+ Tregs was not increased in HNSCC when compared to benign tumors [16].

In a multitude of studies accumulation of tumor-infiltrating CD45RO+ memory T lymphocytes was found to be associated with a favorable clinical outcome in solid tumors. A meta-analysis by Hu et al. concluded that CD45RO+ T cells are a “valuable biomarker for prognostic prediction of human solid malignancies”. Their positive prognostic value has been demonstrated for colorectal cancer (CRC), hepatocellular cancer (HCC), gastric cancer (GC), esophageal cancer (EC), and oral squamous cell carcinoma (OSCC) [12,17,18,19]. In CRC, CD45RO+ T cells strongly correlated with tumor downstaging and long-term outcome after neoadjuvant hypofractionated radiotherapy, indicating a possible role for memory T cells in patient stratification and detection of the most suitable candidates for radiotherapy. Wang et al. hypothesized that increased tumoral antigen release and thus increased memory T cell density might correlate with increased radiosensitivity and that both effects might mutually reinforce each other over the course of radiotherapy treatment [20]. The aim of the present study was to investigate the relationship between CD45RO+ TIL infiltration and long-term survival in HNSCC following radiochemotherapy while focusing on clinical characteristics and treatment modalities. 

## 2. Materials and Methods

We analyzed tumor tissue of 306 consecutively treated patients with locally advanced head and neck cancer (Table 1). The majority of patients suffered from UICC stage IV disease. 

Data on the prognostic value of CD8+ and FoxP3+ T cells in this cohort have been published previously [21].

We used treatment-naïve paraffin-embedded resection specimens or biopsies, which were fixed with formalin, from 306 consecutive patients with locally advanced squamous cell carcinoma of the head and neck.

**Figure 1:**
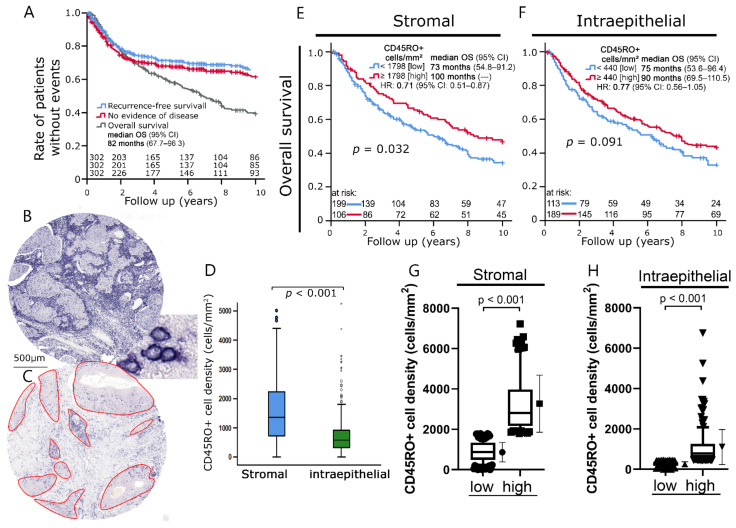
(**A**) Kaplan–Meier plots of 10-year survival of the analyzed cohort divided into overall survival, recurrence-free survival, and no evidence of disease. (**B**) Representative image of a tissue spot from a tissue microarray (TMA) with distinct stromal CD45RO+ cell infiltration. The bottom right window depicts examples of CD45RO+ cells stained by immunohistochemistry. (**C**) Representative image of a tissue spot with weak stromal infiltration. Intraepithelial compartment highlighted by image analysis software. (**D**) Boxplots of stromal and intraepithelial CD45RO+ cell densities in head and neck tumors. The black horizontal line between the two boxplots indicates significant differences between mean values. (**E**,**F**) Kaplan–Meier plots of overall survival according to CD45RO+ cell densities in the (**E**) stromal and (**F**) intraepithelial compartment. (**G**,**H**) Boxplots of CD45RO+ cell density in prognostic groups of Kaplan–Meier curves for overall survival in the (**G**) stromal compartment and (**H**) intraepithelial compartment. Student’s *t*-test was used to compare groups.

The samples were processed into 2 mm diameter tissue microarrays (TMAs). Sections thereof were stained by immunohistochemistry using a monoclonal mouse anti-human CD45RO antibody (DAKO Denmark A/S, Glostrup, Denmark) (Figure 1A). CD45RO+ cells were interpreted as memory T cells. After removal of non-bound antibodies by washing with 50 mM Tris(hydroxymethyl)aminomethan-buffer pH 7.6 supplemented with 0.1% Tween 20, primary antibody was marked by biotinylated horse anti-mouse IgG secondary antibody. Sections were further processed using the VectaStain ABC kit according to the manufacturer’s instructions and DABImmpact (both Vector Laboratories) as substrate. Finally, the sections were counterstained in hemalaun, dehydrated, and covered with Entellan (Merck KGaA, Darmstadt, Germany).

Stained samples were scanned using a whole slide scanner (Axio imager Z2, Zeiss, Göttingen, Germany) at 400× magnification and analyzed using the image analysis software COUNT (Biomas Software, version 3.3; MSAB, Erlangen, Germany). Stromal and intraepithelial compartments were analyzed separately and the respective cell densities were calculated semi-automatically using BIOMAS (Figure 1B). In addition to manually identifying individual cells, the semi-automatic evaluation also included a statistical counting function for heavily infiltrated areas.

Time to death (overall survival), time to local recurrence (recurrence-free survival), and time to local recurrence or distant metastasis (no evidence of disease) were measured in survival analysis.

We performed statistical analyses using SPSS version 26 (IBM Inc., Chicago, IL, USA). Survival plots were generated using the Kaplan–Meier method. X-tile software was used to determine optimal cut-off values for prognostic groups with low and high CD45RO+ cell infiltration (Yale School of Medicine, New Haven, CT, USA). A unique optimal cut-off point was calculated for each Kaplan–Meier plot.

Log-rank test was used to compare survival distributions. Student’s *t*-test for independent samples and Welch’s test were used to compare lymphocyte density distributions. Chi-squared test was used to identify correlations between treatment modalities, clinical characteristics, and cell densities. *p*-values < 0.05 were considered to be statistically significant. 

Cox proportional hazards model was used to calculate hazard ratios of cell densities and clinicopathological characteristics. Covariates with *p* < 0.20 in univariate analysis were included in multivariate analyses. The proportional hazards assumption was verified by visual examination of the log-minus-log curves.

## 3. Results

### 3.1. Patient Cohort

Sections of TMA-embedded tumor samples from a cohort of 306 patients with squamous cell carcinoma of the head and neck were analyzed for CD45RO+ cell density. In most cases, the cancer was advanced, with 195 patients (64%) suffering from UICC stage IV disease (Table 1). After 10 years, overall survival (OS) was 40%, while recurrence-free survival (RFS) and no evidence of disease (NED) status were 65% and 61%, respectively (Figure 1A). CD45RO+ cell densities were registered in the stromal and intraepithelial compartment separately after measurement of the size of the respective areas (Figure 1B,C).

### 3.2. Site and Prognostic Value of CD45RO+ Cell Densities

Stromal CD45RO+ cell densities (median: 1360 CD45RO+ cells/mm^2^) were clearly higher than those in the intraepithelial compartment (median: 572 CD45RO+ cells/mm^2^; *p* < 0.05) (Figure 1D). A high density of stromal CD45RO+ cells was associated with significantly improved 10-year OS (*p* = 0.032) (Figure 1E). High intraepithelial CD45RO+ cell densities likewise tended to be associated with improved survival (*p* = 0.091) (Figure 1F). The distribution of CD45RO+ cell densities in the respective low and high CD45RO+ cell density groups for overall survival defined in Figure 1E,F was analyzed. Both groups were characterized by a large variance in lymphocyte density (Figure 1G,H).

**Figure 2:**
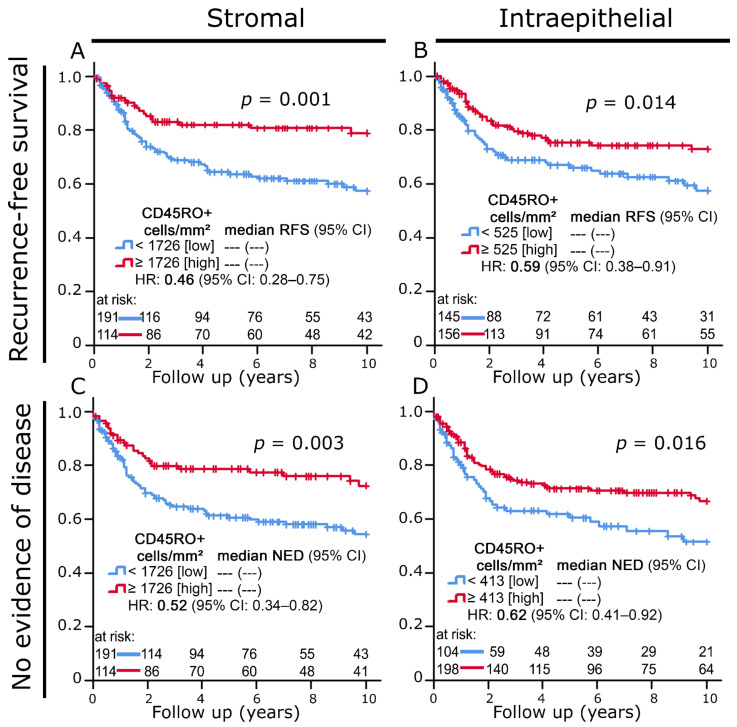
(**A**,**B**) Kaplan–Meier plots of recurrence-free survival according to CD45RO+ cell densities in the (**A**) stromal and (**B**) intraepithelial compartment. (**C**,**D**) Kaplan–Meier plots of no evidence of disease status according to CD45RO+ cell densities in the (**C**) stromal and (**D**) intraepithelial compartment.

Increased stromal and intraepithelial infiltration with CD45RO+ cells also correlated with clearly prolonged recurrence-free survival and no evidence of disease (Figure 2A–D).

### 3.3. Prognostic Significance of CD45RO+ Cell Densities According to Clinical Characteristics

**Figure 3:**
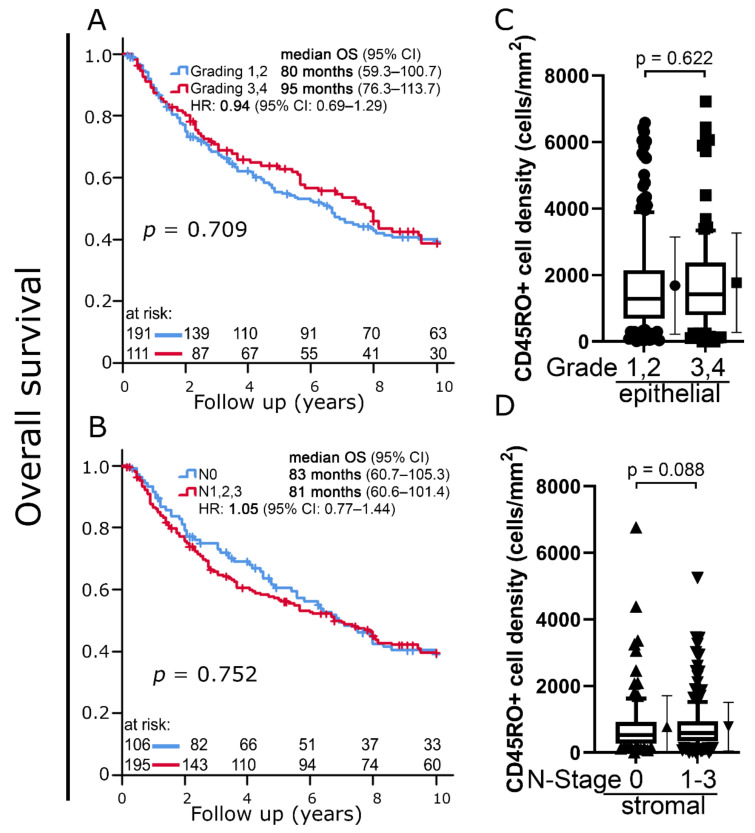
(**A**,**B**) Kaplan–Meier plots of 10-year overall survival according to (**A**) histologic grading and (**B**) lymph node status. (**C**) Boxplots of CD45RO+ cell densities in the intraepithelial compartment according to histologic grading. (**D**) Boxplots of CD45RO+ cell densities in the stromal compartment according to lymph node status. Student’s *t*-test was used to compare groups.

Histologic grading (G1, G2 vs. G3, G4) (*p* = 0.709) as well as lymph node status (N0 vs N1, N2, N3) (*p* = 0.752) were not associated with 10-year OS (Figure 3 A,B).

The distribution of CD45RO+ cell densities did not differ between tumors with low and high histological grading (*p* = 0.622) (Figure 3C). There were also no significant differences between tumors with and without lymph node involvement (*p* = 0.088) (Figure 3D).

**Figure 4:**
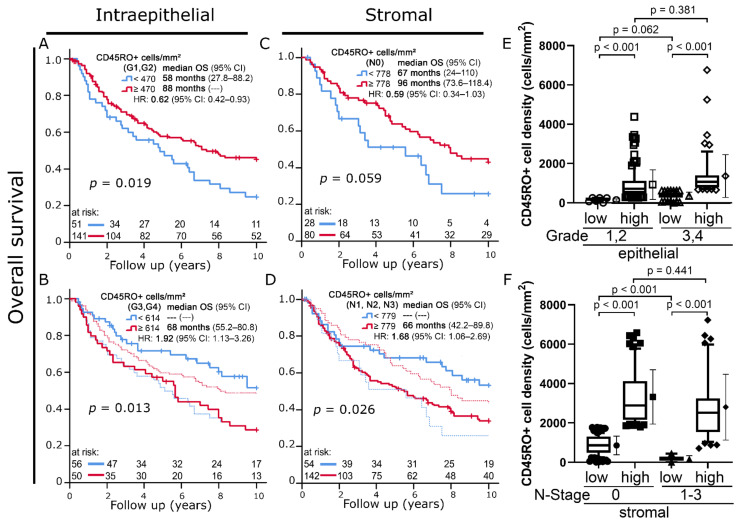
(**A**,**B**) Kaplan–Meier plots of 10-year overall survival according to intraepithelial CD45RO+ cell densities in (A) low-grade (G1, G2) versus (**B**) high-grade (G3, G4) tumors. (**C**,**D**) Kaplan–Meier plots of 10-year overall survival according to stromal CD45RO+ cell densities in patients with (**C**) no lymph node involvement (N0) versus patients with (**D**) positive lymph node status (N1-3). To allow a direct comparison, faded versions of the respective survival curves from (**A**,**C**) were included in (**B**,**D**). (**E**) Boxplots of intraepithelial CD45RO+ cell densities in the prognostic groups defined in (**A**,**B**). (**F**) Boxplots of stromal CD45RO+ cell densities in the prognostic groups defined in (**C**,**D**). Student’s *t*-test was used to compare groups.

In carcinomas with low histologic grading (G1, G2), a high intraepithelial CD45RO+ cell density was once again associated with improved OS (45% vs. 25% 10-year OS; *p* = 0.019) (Figure 4A). In contrast to this, increased intraepithelial infiltration with CD45RO+ cells was a strong indicator of decreased 10-year OS in cancers with high histologic grading (G3, 4) (29% vs. 52% 10-year OS; *p* = 0.013; Figure 4B).

For stromal CD45RO+ cell densities, this ambivalent prognostic value with respect to histologic grading was not found. Instead, a similar phenomenon could be observed when we compared patients with and without lymph node infiltration. In cancers with negative lymph node status (N0, n = 106), high densities of stromal CD45RO+ cells tended to be positively associated with overall survival (10-year OS 43% vs. 26%; *p* = 0.059) (Figure 4C). This prognostic association was inversed when tumors with lymph node infiltration (N1-3; n = 194) were analyzed, and an association with reduced overall survival was found (10-year OS: 34% vs. 53%; *p* = 0.026) (Figure 4D).

Despite their opposite prognostic significance, there was no clear difference in terms of cell densities between the high CD45RO+ cell density group in low-grade tumors (Figure 4A, red) compared to the one in high-grade tumors depicted in Figure 3B (*p* = 0.381) (Figure 3E). The same can be stated when comparing the high CD45RO+ cell density group in N0 tumors (Figure 4C, red) to the high cell density group in N1-3 tumors (*p* = 0.441) (Figure 4F).

We observed no reversal of prognostic value regarding tumor size. Increased CD45RO+ cell densities were non-significantly associated with prolonged 10-year OS in small primary tumors (T1, T2) (stromal: *p* = 0.257; epithelial: *p* = 0.178), as well as T3 and T4 tumors (stromal: *p* = 0.069; epithelial: *p* = 0.064) (Figure S1). The number of patients with distant metastasis was too low (n = 5) to draw a conclusion about a possible change in prognostic significance of CD45RO+ cell density.

In Cox regression analysis, stromal CD45RO+ cell density was an independent and highly significant prognostic factor regarding 10-year overall survival (Table 2).

### 3.4. Predictive Significance of CD45RO+ Cell Density in Patients with Different Treatment Modalities

Patients treated with definitive and neoadjuvant radiochemotherapy (RCT) had significantly more advanced disease stages regarding tumor size, nodal status, and UICC stage than those treated with adjuvant RCT. Stromal CD45RO+ cell density was also increased in these tumors (Table 3).

**Figure 5:**
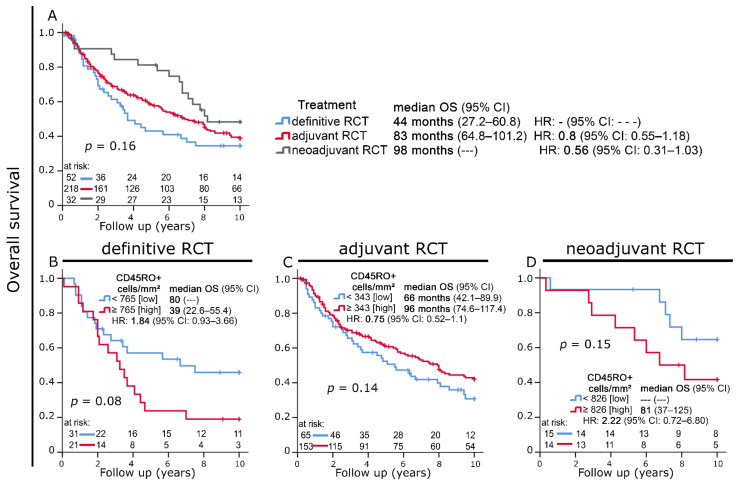
(**A**) Kaplan–Meier plot of overall survival according to treatment modality. (**B**–**D**) Kaplan–Meier plots of overall survival according to intraepithelial CD45RO+ cell densities in patients treated with (**B**) definitive radiochemotherapy (RCT), (**C**) adjuvant RCT, and (**D**) neoadjuvant RCT.

Patients receiving definitive RCT had the shortest median OS time (44 months), while those treated with adjuvant (83 months) and neoadjuvant RCT (98 months) had longer median OS times. This difference, however, was not statistically significant (Figure 5A). We also observed strong trends regarding the predictive significance of CD45RO+ cell densities in different treatment modalities: Increased intraepithelial infiltration with CD45RO+ cells was predictive of decreased OS in patients receiving definitive and neoadjuvant RCT, while it was a positive prognostic factor in the cohort treated with adjuvant RCT (Figure 5B–D).

## 4. Discussion

In the development of new cancer therapies, targeted therapy approaches, such as immune checkpoint inhibitors, play an increasingly important role alongside classical elements such as surgery, chemotherapy, and radiotherapy [22,23]. The goal of this development is not only improved therapeutic efficacy, but more importantly also a reduction of potentially lethal side effects, which commonly include hemato- and nephrotoxicity and can even lead to the occurrence of secondary tumors [24,25]. In this quest, further insights into the influence of various tumor-infiltrating immune cells on the survival of cancer patients are indispensable and, in addition to their role as prognostic factors, could possibly even serve as the groundwork for a new therapeutic approach.

The prognostic value of memory T cells has been reported in various malignancies. In luminal breast cancer, an increased stromal CD45RO+ cell density was a positive prognostic factor and correlated with prolonged disease-free survival [10,26]. In other solid tumors, e.g., colorectal carcinoma, hepatocellular carcinoma, gastric cancer, and esophageal cancer, similar observations regarding the positive prognostic significance of memory T cells were made [12,27,28,29]. A meta-analysis by Hu et al. confirmed this and emphasized the prognostic value of CD45RO+ memory T cells in solid tumors [12]. Concerning head and neck squamous cell carcinomas (HNSCCs), previous studies have reported findings for a wide spectrum of T cell subsets such as CD8+ cytotoxic T cells, CD4+ T helper cells, and FoxP3+ regulatory T cells. Camacho et al. reported on TILs in general by measuring mRNA expression of CD45 through real-time PCR. [21,30,31,32]. CD45RO+ TILs in oral squamous cell carcinoma have been investigated multiple times and were unanimously described as a positive prognostic factor [17,18,19].

In this work, we studied the degree of stromal and intraepithelial infiltration of CD45RO+ memory T cells in head and neck squamous cell carcinomas. We were interested in the relationship between CD45RO+ cell densities and long-term prognosis with a special focus on the influence of clinical characteristics on this association. A clear association between increased CD45RO+ lymphocyte densities and favorable prognosis was found when studying the whole cohort, confirming the results of previous studies.

If a causal relationship is assumed, there are several possible explanations for the commonly reported phenomenon of improved prognosis in connection with memory T cell infiltration. Hu et al. explained the mechanism of action as follows: CD45RO+ T cells are among the major players in adaptive immune defense, which are easily activated and can proliferate and exist throughout life even with low levels of co-stimulation [12,33]. On reoccurrence of tumor antigens in situ, a potent and fast immune response can be executed by effector memory T cells. Another theory related to the post-tumor resection state is that a subset of CD45RO+ memory T cells retract to secondary lymphoid organs, where they exert systemic antitumor effects via various mechanisms, such as INF-γ production, which can in turn improve survival [12].

Interestingly, a closer look at clinical tumor characteristics of the studied cohort revealed that this positive prognostic effect of CD45RO+ TILs was exclusive to low-risk disease stages. In tumors with poor differentiation (histologic grade 3, 4) or local lymph node involvement, the opposite could be observed, and increased CD45RO+ cell infiltration was indicative of reduced overall survival. Moreover, high CD45RO+ cell densities also had a negative predictive significance in patients who were treated with neoadjuvant and definitive radiochemotherapy, both of which correlated strongly with more advanced disease stages.

Tumor-infiltrating memory T cells as a negative prognostic factor are uncommon but not unheard of, as a study by Hotta et al. in renal cell carcinoma reported [34]. However, in their case, increased memory T cell infiltration correlated with more aggressive tumor histology and advanced TNM stage, providing a convincing explanation for reduced survival.

Here, it is noteworthy that there were no significant differences in memory T cell densities between the above-mentioned low- and high-risk groups for both histologic grade and lymph node invasion. Moreover, despite different cut-off values for the high cell density prognostic groups in low-risk versus high-risk disease, the cell density distribution in these two subgroups was also comparable. This clearly indicates that increased infiltration with CD45RO+ memory T cells can have a completely opposite prognostic predictive value. On a mechanistic level, once more under the assumption of a causal relationship, this means that in advanced tumors, modifications of the TME must occur, leading to this reversal of prognostic relevance. A possible explanation for this observation could be that a tumor which moves further away from its original differentiation also undergoes a significant change in antigens presented to immune cells. This could, despite large accumulations of effector cells, render the highly specific subgroup of memory T cells functionally ineffective. Another factor could be oxidative stress, which increases with the progression of the tumor. As shown by Malmberg et al., activated CD45RO+ subsets are more vulnerable to oxidative stress than naïve cells [35]. Furthermore, more advanced and aggressive tumor stages might lead to an increase in inflammatory factors in the TME. Inflammation can be a powerful modulator of the immune response to a tumor. As we reported previously in HNSCC and rectal cancer, the prognostic relevance of FoxP3+ regulatory T cells can completely change in the presence of cellular inflammation [21,36]. A similar mechanism could be an underlying cause of the reversal of prognostic association we observed here, with increased inflammation in more advanced tumors altering the function of memory T cells.

An additional aspect to consider is heterogeneity within the group of CD45RO+ cells, since the expression of CD45RO is not exclusive to activated memory T cells [37]. Other CD45RO-expressing cells are B lymphocytes as well as natural killer cells [10,37,38,39]. Both have been described as a positive prognostic factor in HNSCC [40,41]. Additionally, as much as 90–95% of FoxP3+ regulatory T cells (Tregs) in adult blood express CD45RO [42]. While tumor-infiltrating regulatory T cells are classically seen as an immunosuppressive and thus prognostically unfavorable element in HNSCC, T reg infiltration has also been associated with prolonged survival [16,21,43].

All these inflammatory cell types fall into the complex spectrum of CD45RO+ TILs, and it is not possible to distinguish between them in a single marker investigation. Advanced tumor stages in our study could for example be infiltrated by a higher percentage of suppression competent Tregs contributing to disease progression. Studies using multiple immunohistochemical markers should therefore be conducted in the future to further characterize CD45RO+ TILs in different disease stages.

A major strength of the study we conducted was the high degree of precision that was achieved by counting stained cells manually and separately in the stromal and intraepithelial compartments. Furthermore, we studied a relatively large cohort of over 300 patients and had long-term survival data of at least 10 years for most patients.

Weaknesses were the retrospective design, an uneven distribution in terms of gender ratio, tumor location, and treatment modalities, all of which might have had an influence on the relationship between memory T cell infiltration and survival. Moreover, we only measured cell densities in one TMA spot per patient. For this reason, our results might not fully reflect intratumoral heterogeneity in cell densities. In general, however, there is a very high degree of concordance between TMA spots and whole tissue sections in immunohistochemical studies [44]. Data on some clinical characteristics were not available for all patients. Another important fact to reiterate at this point is that our study only allowed us to observe associations, making all hypotheses on causal relationships purely speculative.

## 5. Conclusions

Clinical characteristics must be considered when evaluating tumor-infiltrating memory T cells for their prognostic and predictive significance in radiochemotherapy of HNSCC. Increased infiltration with CD45RO+ cells was only indicative of prolonged survival in low-risk tumors without local lymph node involvement or with low histological grade. In contrast to this, high CD45RO+ TIL densities were associated with reduced overall survival in more advanced cases. Therefore, future studies on tumor-infiltrating memory T cells in other cancer entities should also be conducted under careful consideration of clinical factors.

## Figures and Tables

**Table 1:** Clinical characteristics of the studied patient cohort.

Clinical Characteristics	(Number of Patients) Percentage
Sex	male (258) 84%
	female (48) 16%
Median age (years)	55 (SD: 9.16, min: 27, max: 81)
Location/primary site of the tumor	throat, oro-, naso-, hypopharynx, base of tongue (237) 77%
	floor of mouth (8) 3%
	palate (61) 20%
UICC Stage	I (13) 4%
	II (28) 9%
	III (56) 18%
	IV (209) 68%
Primary tumor	T1 (58) 19%
	T2 (102) 33%
	T3 (74) 24%
	T4 (72) 24%
Pathological regional lymph nodes	N0 (108) 35%
	N1 (38) 12%
	N2 (134) 44%
	N3 (26) 8%
Distant metastasis	M0 (301) 98%
	M1 (5) 2%
Grading	G1 (16) 5%
	G2 (178) 58%
	G3 (104) 34%
	G4 (8) 3%
Treatment	0 def. RCT (52) 17%
	1 OP + adj. RCT (222) 73%
	2 Neoadj. RCT + OP (32) 10%

SD = standard deviation, min = minimum age, max = maximum age, UICC = Union for International Cancer Control, def. = definitive, RCT = radiochemotherapy, OP = operation, adj. = adjuvant, neoadj. = neoadjuvant.

**Table 2:** Univariate and multivariate analysis of overall survival according to the Cox proportional hazards model.

	Univariate Analysis			Multivariate Analysis		
Variable	Hazard Ratio	95% C.I.	*p*	Hazard Ratio	95% C.I.	*p*
Age (year) (<55 (n = 150) vs. ≥55 (n = 148))	1.083	0.802–1.461	0.60	---	---	---
Sex (Male (n = 252) vs. Female (n = 46))	0.884	0.574–1.361	0.58	---	---	---
T Stage (T1, T2 (n = 155) vs. T3, T4 (n = 143))	1.244	0.922–1.68	0.15	1.144	0.816–1.605	0.43
N Stage (N0 (n = 106) vs. N1, N2, N3 (n = 192))	1.052	0.767–1.443	0.75	---	---	---
Histological grading (G1, G2 (n = 188) vs. G3, G4 (n = 110))	0.943	0.69–1.288	0.71	---	---	---
Surgery (No (n = 52) vs. Yes (n = 246))	0.765	0.523–1.119	0.17	0.756	0.517–1.107	0.15
Stromal CD45RO+ cell density (low (n = 193) vs. high (n = 105 ))	0.706	0.512–0.974	**0.03**	0.698	0.504–0.965	**0.03**
Intraepithelial CD45RO+ cell density (low (n = 113) vs. high (n = 185))	0.768	0.564–1.045	0.09	0.823	0.6–1.129	0.23

**Table 3:** Distribution of clinical characteristics and CD45RO+ cell density according to treatment modality.

		Treatment			
	N (Total)	Definitive RCT	Adjuvant RCT	Neoadjuvant RCT	*p*
**Age (year)**					0.2
<55	155	22 (42%)	113 (51%)	20 (63%)	
≥55	151	30 (58%)	109 (49%)	12 (37%)	
**Sex**					0.68
male	258	42 (81%)	188 (85%)	28 (88%)	
female	48	10 (19%)	34 (15%)	4 (12%)	
**T Stage**					**<0.001**
T1,2	160	3 (6%)	145 (65%)	12 (38%)	
T3,4	146	49 (94%)	77 (35%)	32 (62%)	
**N Stage**					**<0.001**
N0	108	8 (15%)	98 (44%)	2 (7%)	
N1,2,3	197	44 (85%)	124 (56%)	29 (93%)	
**UICC Stage**					**<0.001**
I, II, III	97	6 (12%)	85 (38%)	6 (19%)	
IV	209	46 (89%)	137 (62%)	26 (81%)	
**Histological grading**					0.17
G1,2	194	36 (69%)	134 (60%)	24 (75%)	
G3,4	112	16 (31%)	88 (40%)	8 (25%)	
**Stromal CD45RO+ cell density**					0.94
low	199	35 (67%)	144 (65%)	20 (65%)	
high	106	17 (33%)	78 (35%)	11 (35%)	
n.a.	1				
**Intraepithelial CD45RO+ cell density**					**0.003**
low	114	17 (33%)	94 (42%)	3 (10%)	
high	189	35 (67%)	128 (58%)	26 (90%)	
n.a.	3				

RCT = radiochemotherapy, UICC = Union for International Cancer Control, n.a. = not available.

## Data Availability

The data presented in this study are available on request from the corresponding author.

## Supplementary Materials

The following supporting information can be downloaded at: https://www.mdpi.com/article/10.3390/cells11121960/s1, Figure S1: (A,B) Kaplan–Meier plots of 10-year overall survival according to stromal CD45RO+ cell densities in patients with T1, T2 tumors versus patients with (B) T3, T4 tumors. (C,D) Kaplan–Meier plots of 10-year overall survival according to intraepithelial CD45RO+ cell densities in patients with T1, T2 tumors versus patients with (D) T3, T4 tumors.
