# Supplementary figures and images for: The Prognostic and Predictive Significance of Tumor-Infiltrating Memory T Cells Is Reversed in High-Risk HNSCC
Source: Cells. 2022 Jun 17;11(12):1960. doi: 10.3390/cells11121960 (PMC9221945; doi:10.3390/cells11121960)

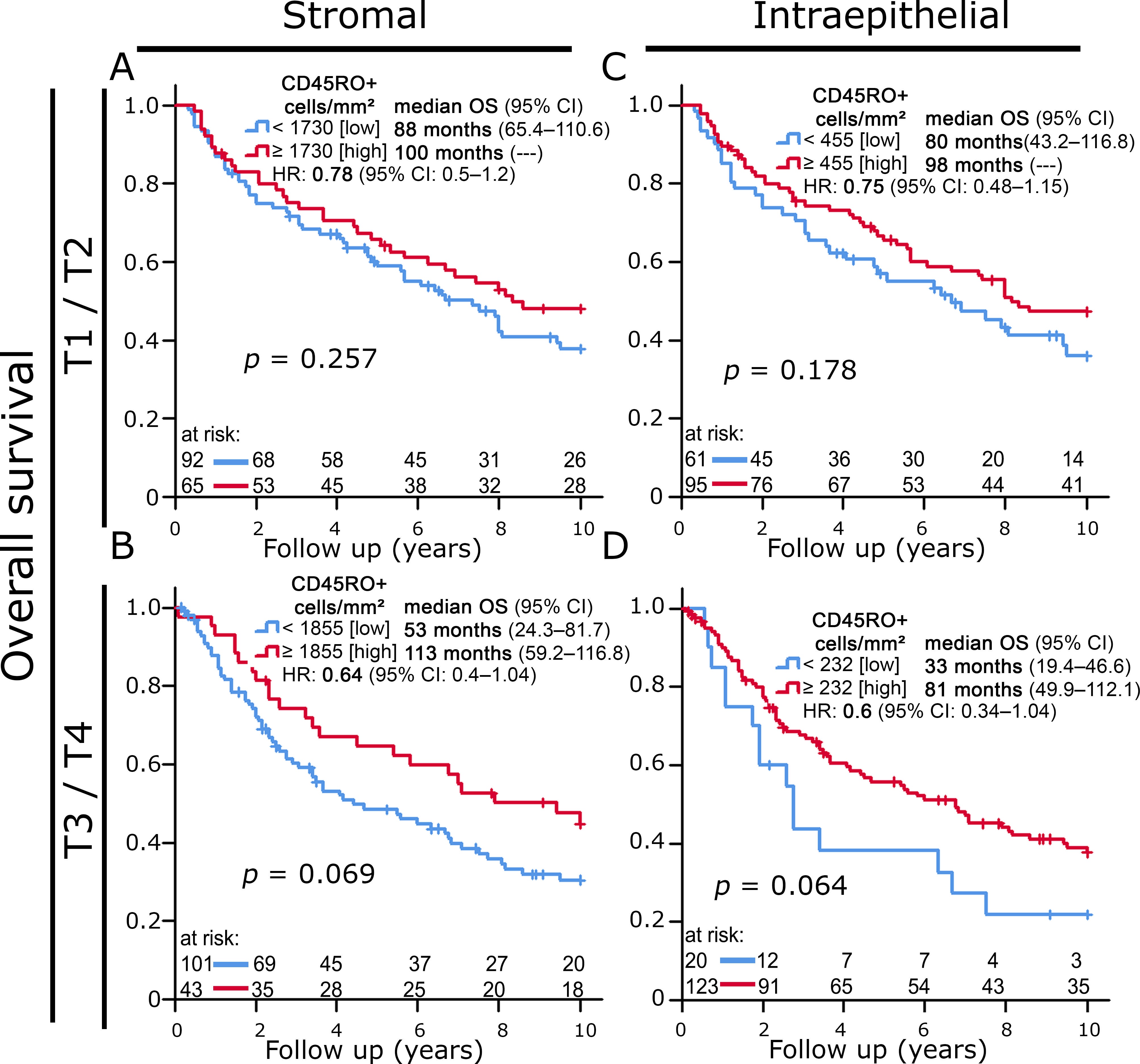

Supplement: Supplementary file 1 [file cells-11-01960-s001.zip › cells-1756772-supplementary.png]
